# A frameshift mutation in *ARMC3* is associated with a tail stump sperm defect in Swedish Red (*Bos taurus*) cattle

**DOI:** 10.1186/s12863-016-0356-7

**Published:** 2016-02-29

**Authors:** Hubert Pausch, Heli Venhoranta, Christine Wurmser, Kalle Hakala, Terhi Iso-Touru, Anu Sironen, Rikke K. Vingborg, Hannes Lohi, Lennart Söderquist, Ruedi Fries, Magnus Andersson

**Affiliations:** Lehrstuhl fuer Tierzucht, Technische Universitaet Muenchen, 85354 Freising, Germany; Department of Production Animal Medicine, Faculty of Veterinary Medicine, University of Helsinki, 04920 Saarentaus, Finland; Natural Resources Institute Finland (Luke), Green Technology, 31600 Jokioinen, Finland; Genoskan A/S, 8830 Tjele, Denmark; Department of Veterinary Biosciences and Research Programs Unit, Molecular Neurology, University of Helsinki and Folkhälsan Research Center, 00290 Helsinki, Finland; Division of Reproduction, Department of Clinical Sciences, Swedish University of Agricultural Sciences, SE-750 07 Uppsala, Sweden

**Keywords:** ARMC3, Tail stump sperm defect, Swedish Red cattle, MMAF, Flagellum, Male infertility, Spermatogenesis

## Abstract

**Background:**

Artificial insemination is widely used in many cattle breeding programs. Semen samples of breeding bulls are collected and closely examined immediately after collection at artificial insemination centers. Only ejaculates without anomalous findings are retained for artificial insemination. Although morphological aberrations of the spermatozoa are a frequent reason for discarding ejaculates, the genetic determinants underlying poor semen quality are scarcely understood.

**Results:**

A tail stump sperm defect was observed in three bulls of the Swedish Red cattle breed. The spermatozoa of affected bulls were immotile because of severely disorganized tails indicating disturbed spermatogenesis. We genotyped three affected bulls and 18 unaffected male half-sibs at 46,035 SNPs and performed homozygosity mapping to map the fertility disorder to an 8.42 Mb interval on bovine chromosome 13. The analysis of whole-genome re-sequencing data of an affected bull and 300 unaffected animals from eleven cattle breeds other than Swedish Red revealed a 1 bp deletion (Chr13: 24,301,425 bp, ss1815612719) in the eleventh exon of the armadillo repeat containing 3-encoding gene (*ARMC3*) that was compatible with the supposed recessive mode of inheritance. The deletion is expected to alter the reading frame and to induce premature translation termination (p.A451fs26). The mutated protein is shortened by 401 amino acids (46 %) and lacks domains that are likely essential for normal protein function.

**Conclusions:**

We report the phenotypic and genetic characterization of a sterilizing tail stump sperm defect in the Swedish Red cattle breed. Exploiting high-density genotypes and massive re-sequencing data enabled us to identify the most likely causal mutation for the fertility disorder in bovine *ARMC3*. Our results provide the basis for monitoring the mutated variant in the Swedish Red cattle population and for the early identification of infertile animals.

**Electronic supplementary material:**

The online version of this article (doi:10.1186/s12863-016-0356-7) contains supplementary material, which is available to authorized users.

## Background

Artificial insemination (AI) is widely used instead of natural mating in many cattle breeding populations. Ejaculates of breeding bulls are collected once or twice a week and closely examined immediately after semen collection at highly specialized AI centers. Only ejaculates without apparent abnormalities are retained for AI. Up to 20 % of all collected ejaculates are rejected because they do not comply with current standards for AI [1]. Diagnoses of insufficient semen quality involve the absence of spermatozoa, low sperm concentration, reduced motility or viability and morphological aberrations of spermatozoa [2].

A motile sperm flagellum is essential for the fertilization *in vivo*. Morphological aberrations of the sperm tail compromise sperm motility and impair fertilization. Such aberrations are collectively referred to as multiple morphological abnormalities of the flagella (MMAF, [3]). Diagnoses of MMAF involve stump and short tail spermatozoa and dysplasia of the fibrous sheath. Sequence variants causing MMAF have been identified in, *e.g.*, humans [3–5], pigs [6, 7] and mice [8–10]. However, sequence variants causing MMAF have not been identified in cattle so far.

Bulls with MMAF have been observed in Holstein-Friesian, Ayrshire and Indobrasil cattle [11–15]. The affected bulls were isolated cases within their breeds without known relationship among each other indicating a heterogeneous genetic etiology of MMAF across breeds. However, Alanko et al. [16] reported three related bulls from the Ayrshire cattle breed with a sterilizing tail stump sperm defect suggesting that such conditions may be inherited in an autosomal recessive fashion in cattle.

Here we present the phenotypic manifestation and the genetic analysis of a recessively inherited tail stump sperm defect in the Swedish Red cattle breed. The application of homozygosity mapping facilitated the mapping of the fertility disorder to a short segment on bovine chromosome 13. The analysis of comprehensive whole-genome sequence data revealed a frameshift mutation in *ARMC3* that most likely causes the sperm tail disorder in Swedish Red cattle.

## Results

### A recessively inherited tail stump sperm defect in the Swedish Red cattle breed

**Fig. 1:**
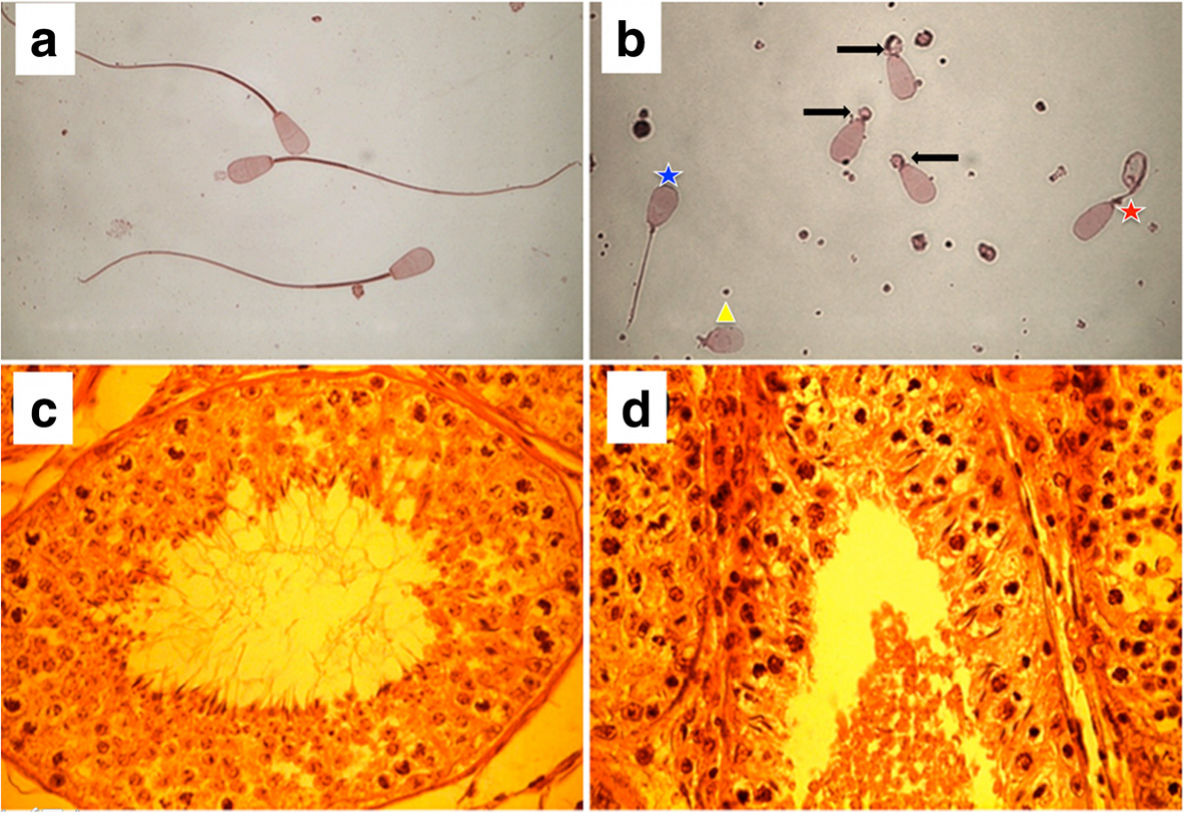
Phenotypic manifestation of the tail stump sperm defect. Representative figures of spermatozoa of a control (**a**) and an affected bull (**b**). Spermatozoa of affected bulls had multiple aberrations such as short tails (blue star), rudimentary tails with proximal droplet (arrows), rudimentary tails without proximal droplet (yellow triangle) and coiled tails (red star). Histological sections of the testicles of a control (**c**) and an affected (**d**) bull. Numerous full-length sperm tails are present in the luminal part of the tubuli seminiferi in the control bull, whereas full-length sperm tails are absent in the affected bull

Three young bulls (11 months) of the Swedish Red cattle breed born in 2008, 2009 and 2012, were reported from an AI center because they produced ejaculates with immotile spermatozoa during a semen collection period of 5 months. Examination of the bulls’ fresh ejaculates revealed a reduced sperm concentration (~140 million spermatozoa per ml) despite normal ejaculate volume (~4 ml). The sperm count was only 10–20 % of the average sperm count of control bulls. All spermatozoa were immotile because of multiple flagellar abnormalities such as rudimentary (less than 5 % of the normal length), short length and absent tails. A proximal droplet surrounded most rudimentary tails (Fig. 1a-b). The proportion of spermatozoa with abnormal heads ranged from 47 to 62 %, which is ten times higher than in normal ejaculates (Table 1). None of the spermatozoa were motile. Histological sections of the testicles revealed a lack of full-length sperm tails in the luminal part of the tubuli seminiferi indicating disturbed spermatogenesis (Fig. 1c-d).

**Table 1:** Sperm morphology in fresh ejaculates of three affected AI bulls

Phenotype		Bull 1	Bull 2	Bull 3
Tail morphology	Normal tails	0 %	0 %	0 %
	Absent tails	2 %	3 %	4 %
	Rudimentary tails	45 %	63 %	28 %
	Short straight tails	27 %	15 %	29 %
	Folded or coiled short tails	26 %	19 %	39 %
Head morphology	Normal heads	42 %	53 %	38 %
	Abnormal heads	58 %	47 %	62 %

The analysis of the pedigree records of three affected bulls revealed a common ancestor (born in 1987) in their paternal and maternal path (see Additional file 1). Eighteen male half-sibs of the affected bulls were used for AI. The quality of their ejaculates was normal and their fertility records were within reference ranges indicating undisturbed reproductive performance. Based on these findings, an autosomal recessive mode of inheritance was assumed for the tail stump sperm defect.

### The tail stump sperm defect maps to bovine chromosome 13

**Fig. 2:**
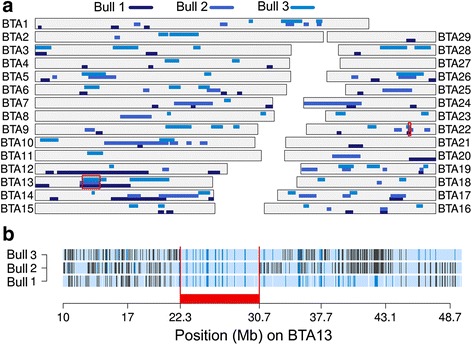
Homozygosity mapping in three animals with a sterilizing tail stump sperm defect. **a** Shades of blue represent long runs of homozygosity (ROH) in three animals along the 29 autosomes. The red borders highlight two regions on BTA13 and BTA22 with ROH in all affected animals. **b** Autozygosity mapping on BTA13 in three affected animals. Blue and pale blue represent homozygous genotypes (AA and BB), heterozygous genotypes (AB) are displayed in light grey. White color indicates missing genotypes. The red bar indicates a common 8.42 Mb segment of homozygosity

To identify the genomic region associated with the tail stump sperm defect, three affected and 18 unaffected male half-sibs were genotyped with the Illumina BovineSNP50 genotyping array. After quality control, genotypes at 46,035 SNPs were screened for the presence of long runs of homozygosity (ROH) in three affected bulls. Only two genomic regions were consistently homozygous in all affected animals: a 1.13 Mb segment on BTA22 (from 48,349,750 bp to 49,479,051 bp) and an 8.42 Mb segment on BTA13 (from 22,308,682 bp to 30,733,648 bp) (Fig. 2a). The segment on BTA22 was also homozygous in six fertile half-sibs precluding an association with the tail stump sperm defect. In contrast, the 8.42 Mb segment on BTA13 was never found in the homozygous state in eighteen unaffected half-sibs corresponding to an autosomal recessive inheritance (Fig. 2b).

### A 1 bp deletion in *ARMC3* is associated with the tail stump sperm defect

To pinpoint the mutation causing the tail stump sperm defect, the whole genome of an affected bull was sequenced to an average read depth of 9.29. In addition, we exploited data of 300 previously sequenced animals from eleven cattle breeds other than Swedish Red for the identification of the mutation. Deleterious recessive mutations are assumed to have occurred after breed formation and are thus likely to be breed-specific. Thus we assumed that the causal mutation should not segregate among the sequenced control animals. Multi-sample variant calling in the 8.42 Mb region of extended homozygosity on BTA13 yielded genotypes at 81,925 single nucleotide and short insertion and deletion polymorphisms (74,385 SNPs, 7540 Indels). In addition, 11,505 structural variants were detected in the genome-wide sequence data of the affected bull and 226 control animals with genome coverage of at least eight-fold.

**Fig. 3:**
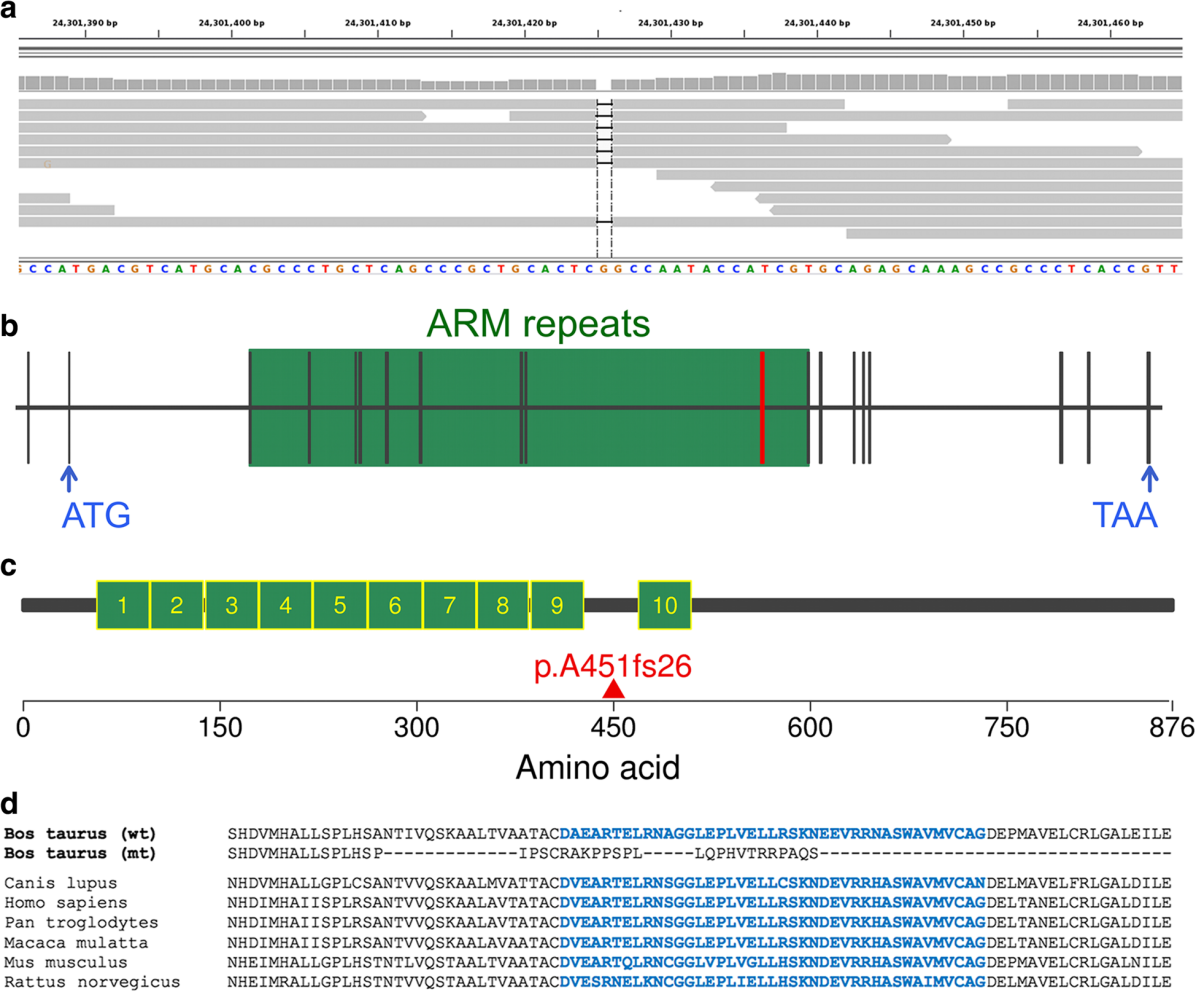
A 1 bp deletion in *ARMC3* induces premature translation termination. **a** Snapshot from the Integrated Genomics Viewer (IGV, [51]) showing a homozygous 1 bp deletion on chromosome 13 at 24,301,425 bp in an animal with the tail stump sperm defect. **b** Genomic structure of bovine *ARMC3*. Bovine *ARMC3* consists of 19 exons (vertical bars) and its translation starts in exon 2. The red vertical bar represents the eleventh exon where the 1 bp deletion is located. The coordinates of ten Armadillo (ARM) repeats were determined using the *Simple Modular Architecture Research Tool* [50]. Blue arrows represent the position of the start and stop codons. **c** The bovine ARMC3 protein sequence consists of 876 amino acids and it contains ten ARM repeats (green boxes). The red triangle represents the start of the shift in translation resulting from the 1 bp deletion. **d** Multi-species alignment of a part of the ARMC3 protein sequence. Blue colour highlights the protein sequence of the tenth ARM repeat, which is absent in the mutated (mt) bovine sequence

Seventy-seven variants were compatible with recessive inheritance that is homozygous for the reference allele in 300 control animals and homozygous for the alternate allele in the affected bull. Bioinformatic analysis revealed that 76 variants were located in non-coding regions of the genome and one variant resided in the coding region of the armadillo repeat containing 3-encoding gene (*ARMC3*, Chr13: 24,301,425 bp, ss1815612719, Fig. 3a, see Additional files 2 and 3).

To further reduce the number of plausible candidate causal mutations, we exploited whole-genome sequence data of 1147 animals from 29 cattle breeds that had been sequenced for Run4 of the 1000 bull genomes project [17]. Because of the close relationship among animals of three Nordic Red cattle breeds, we excluded 56 sequenced animals from the Ayrshire, Swedish Red and Danish Red cattle breed for variant filtering. Thirty-five out of 77 compatible variants also segregated among 1009 animals from breeds other than Nordic Red (see Additional file 4). In conclusion, the coding variant in *ARMC3* and 41 non-coding variants were considered as candidate causal variants for the tail stump sperm defect.

The bovine *ARMC3* gene consists of 19 exons encoding 876 amino acids (Fig. 3b). The variant compatible with recessive inheritance (ss1815612719) is a 1 bp deletion in the eleventh exon of *ARMC3* affecting the third base of codon 450 (ENSBTAT00000061467:c.1350_1351delGGinsG). Sanger sequencing confirmed homozygosity for the deletion variant in two bulls with the tail stump sperm defect. The 1 bp deletion is expected to alter the reading frame and to change the amino acid sequence from position 451 onwards resulting in a premature translation termination at position 476 (p.A451fs26). The mutated protein should be shortened by 401 amino acids (46 %). Bioinformatic analysis revealed that the protein sequence of bovine ARMC3 contains ten armadillo/beta-catenin-like (ARM) repeats (Fig. 3c). The deletion variant resides within the highly conserved armadillo repeat containing domain. Due to the frameshift with premature translation termination, the mutated protein is expected to lack one ARM repeat (Fig. 3d).

We genotyped 97 AI bulls from the Swedish Red cattle breed with normal fertility at ss1815612719 using customized genotyping assays. None of the bulls was homozygous for the deletion variant. Seventy-four bulls were homozygous for the reference allele and 23 bulls were heterozygous carriers of the 1 bp deletion yielding a frequency of the deletion of 11.9 %.

## Discussion

Although there is considerable phenotypic variation both in semen quality and insemination success of AI bulls, the genetic determinants underlying male reproductive traits are scarcely understood [18]. Low heritability of fertility traits and small-sized samples complicated the mapping of causal sequence variants in the past. Moreover, fertility-associated variants did not reach convincing levels of significance in replication studies [19, 20]. Recently, the availability of comprehensive genotype and massive re-sequencing data enabled the identification of a recessively inherited variant of idiopathic male subfertility in cattle [21]. However, to our knowledge, our study is the first to reveal a mutation that manifests in morphological aberrations of the spermatozoa in cattle.

The analysis of pedigree records indicated that the sperm tail disorder is inherited in an autosomal recessive fashion. Sequence variants underlying recessive traits are traditionally identified by comparing allele counts of dense molecular markers in affected and unaffected individuals (*e.g.*, [21]). The likelihood to map a mendelian trait in a genome-wide case/control-association study depends on the number of affected individuals [22]. The tail stump sperm defect is a rare disorder in the Swedish Red cattle breed. Assuming a frequency of the deleterious allele of 12 % in the population, random mating and 100 bulls that are annually purchased by the Swedish AI center, one would expect only one of them to be affected by the tail stump sperm defect. Accordingly, only three affected bulls were recognized in the past 10 years. We genotyped those bulls with a genotyping array and resorted to perform homozygosity mapping, which facilitates pinpointing genomic regions underlying recessive traits with a small number of affected individuals [23]. Three affected bulls had a common 8.42 Mb segment of extended homozygosity which is a typical length observed in studies that are based on few affected animals [23–26]. Compatible with recessive inheritance, none of the fertile half-sibs was homozygous. Next generation sequencing of an affected bull revealed a frameshift mutation in *ARMC3* (ss1815612719, c.1350delG, p.A451fs26) that segregated with the tail stump sperm defect. Forty-one variants in non-protein-coding regions were also associated with the disorder. However, we consider the frameshift in *ARMC3* as the most likely causal mutation because it is predicted to result in a protein that lacks 401 amino acids. The function of the truncated ARMC3 protein may be severely compromised, since it lacks domains that are likely required for normal protein function [27].

Absence or impaired function of ARMC3 possibly prevents physiological spermatogenesis resulting in morphological aberrations of the spermatozoa. The sperm tails of homozygous bulls were severely disorganized and all spermatozoa were immotile precluding successful fertilization *in vivo*. Apart from immotile spermatozoa, the bulls were healthy. The morphological aberrations of the spermatozoa are similar to those observed in the Ayrshire cattle breed [12–14, 16]. Because Swedish Red cattle are closely related to Ayrshire cattle [28, 29], it is possible that the frameshift mutation in *ARMC3* occurred in a common ancestor of the two breeds and that it might also be associated with the sperm tail disorder in Ayrshire cattle. However, the genetic underpinnings of apparently similar phenotypes may be completely different across breeds (*e.g.*, [24, 30, 31]). In any case, it is recommended to survey sequence variants in *ARMC3* in bulls with fertility disorders in cattle breeds other than Swedish Red.

To our knowledge, our study reveals for the first time an association of a mutation in *ARMC3* with morphological abnormalities of the sperm flagellum. However, deleterious mutations in other genes encoding armadillo repeat-containing proteins have already been shown to compromise sperm motility [8, 32]. In our study, the spermatozoa of bulls that were homozygous for the frameshift mutation in *ARMC3* were immotile because of severe flagellar abnormalities. A previous study demonstrated that dysfunction of *ARMC4*, a paralog to *ARMC3*, impairs physiological function of the cilia and sperm flagella in humans [33]. Proper function of *Gudu*, a gene highly homologous to *ARMC4*, is essential for an undisturbed spermatogenesis in *Drosophila melanogaster* [34]. Our investigations also evidenced an impaired spermatogenesis in bulls homozygous for the frameshift mutation in *ARMC3*. Such findings suggest a crucial role of *ARMC3* for physiological spermatogenesis.

The morphological aberrations of the spermatozoa observed in our study are similar to those observed in Yorkshire boars with a loss of function mutation in *SPEF2* [6]. Both defects manifest in immotile spermatozoa precluding fertilization *in vivo* both in natural service and AI. The phenotypic manifestations of the two defects differ only slightly. Spermatozoa of animals being homozygous for the *ARMC3* frameshift mutation mostly lack the midpiece with mitochondria, which is, however, commonly present in spermatozoa of animals homozygous for the *SPEF2* mutation [6].

## Conclusions

The combination of high-density genotype and whole-genome re-sequencing data revealed a recessively inherited frameshift mutation in bovine *ARMC3* that most likely causes a sterilizing tail stump sperm defect in Swedish Red cattle. Our findings suggest that impaired function of ARMC3 compromises spermatogenesis and thereby results in severely disorganized sperm tails, which prevents successful fertilization *in vivo*. Compared to mutations that manifest in idiopathic male sub- or infertility [21], spermatozoa of affected animals have striking morphological aberrations that facilitate to unambiguously identify homozygous bulls at AI centers. However, our findings facilitate to identify affected young bulls before they are purchased by AI centers using *e.g.*, genotyping assays on customized genotyping arrays.

## Methods

### Animal ethics statement

All animals were housed at an approved commercial AI center in Örnsro, Sweden. Semen samples were collected by employees of the AI center as part of their regular breeding and reproductive measures in cattle industry. Bulls with the tail stump sperm defect were slaughtered because their semen was not suitable for artificial insemination. The decision to slaughter the bulls was made solely by the owner (*i.e.*, AI center) of the bulls. None of the authors of the present study was involved in the decision to slaughter the bulls. Testicles of an affected bull were collected after slaughter. Consent from the owner of the bulls was obtained to use the semen and tissue samples for this study. No ethical approval was required for this study.

### Animals

Three bulls of the Swedish Red cattle breed born between 2008 and 2012 with a sterilizing tail stump sperm defect were included in the study together with 18 unaffected fertile male half-sibs. The bulls were housed in an AI bull center in Örnsro, Sweden. The age of the bulls during semen collection ranged from 11 to 16 months. Employees from the AI center collected semen approximately twice a week as part of their regular practice.

### Sperm motility, morphology and testicular histology

We examined ten ejaculates per bull. Aliquots of fresh semen were put into vials to measure sperm concentration using a photometric method and a haemocytometer (Bürker chamber). A drop of semen (approximately 7 μl was put on a pre-warmed slide to evaluate sperm morphology. Head and sperm tail morphology of 200 spermatozoa was assessed from slides stained with the Williams stain (bright field microscopy) and from a wet mount formol-saline sample using a phase contrast microscope with 1000× magnification, respectively. Moreover, sperm head morphology was assessed in dry smears stained with carbol fuchsin according to Williams [35] and Lagerlöf [36]. Testicles from an affected bull were collected after slaughter. Histological specimens were taken from the testicles, fixed in Bouin’s solution and embedded in paraffin. Sections (5 μm) were cut and stained with haematoxylin and eosin.

### Genotyping of affected and unaffected animals

Twenty-one bulls (three affected, 18 unaffected) of the Swedish Red cattle breed were genotyped using the Illumina BovineSNP50 Bead chip (Illumina, Inc., San Diego, CA, USA). The chromosomal position of the SNPs corresponded to the UMD3.1 assembly of the bovine genome [37]. Mitochondrial, X-chromosomal, Y-chromosomal SNPs and SNPs with unknown chromosomal position were not considered for further analyses. After quality control (per SNP and per individual call-rate higher than 90 %, no deviation from the Hardy-Weinberg equilibrium (*P* > 0.0001)), 46,035 SNPs were retained for further analyses. *Beagle* genetic analysis software [38] was used to impute sporadically missing genotypes and to infer haplotypes.

### Homozygosity mapping

Segments of extended homozygosity were identified in three affected bulls using the *homozyg*-function implemented in the whole genome association analysis toolset *PLINK* [39, 40]. Due to the relatively sparse genome coverage of the genotype data (1 SNP per 56 kb), we restricted our analysis to runs of homozygosity (ROH) with a minimum number of 20 contiguous homozygous SNPs and a minimum length of 500 kb.

### Generation of sequence data

Genomic DNA of an affected bull was prepared from a semen sample following standard protocols using proteinase K digestion and phenol-chloroform extraction. A gDNA sequencing library with 420 bp insert size was prepared using the TruSeq DNA Sample Preparation Kit (Illumina inc., San Diego, CA, USA). The sample was sequenced on an Illumina HiSeq2500 system using TruSeq SBS v3 chemistry (Illumina inc., San Diego, CA, USA) and the 2x100 bp paired-end read module. The *fastq*-files were generated with the *CASAVA bcl2fastq* conversion software (version 1.8.3, Illumina inc., San Diego, CA, USA). The alignment of the reads to the University of Maryland reference sequence (UMD3.1, [37]) was performed with the *Burrows-Wheeler Aligner* [41]. The resulting SAM file was converted into a BAM file with *SAMtools* [42]. Duplicate reads were identified and marked with the MarkDuplicates command of *Picard*-tools [43].

### Identification of candidate causal variants

Single nucleotide and short insertion and deletion polymorphisms were genotyped in the affected bull together with 300 previously sequenced animals from eleven cattle breeds (Gelbvieh (*n* = 12), Nordic Finncattle (*n* = 6), Fleckvieh (*n* = 153), Original Simmental (*n* = 15), Holstein-Friesian (*n* = 31), Brown Swiss (*n* = 50), Murnau-Werdenfelser (*n* = 2), Ayrshire (*n* = 2), Red-Holstein (*n* = 21), Original Braunvieh (*n* = 8)) other than Swedish Red [44] using the multi-sample approach implemented in the *mpileup* function of *SAMtools* [42] and a variant calling pipeline as detailed by Jansen et al. [25]. Larger insertions and deletions and structural rearrangements were identified in the affected animal and 226 sequenced control animals with an average genome coverage above 8-fold using the *Pindel* software package [45]. To identify mutations compatible with recessive inheritance, all polymorphic sites were filtered for variants that were homozygous for the alternate allele in the affected bull and homozygous for the reference allele in 300 sequenced control animals. Candidate causal variants were annotated using the *Variant Effect Predictor* tool [46, 47]. Additionally, sequence variants of 1147 animals from 29 breeds that were sequenced for the 1000 bull genomes project [17] were analyzed to obtain genotypes of compatible variants in a larger cohort. The animals of the 1000 bull genomes project were mostly influential sires that had been widely used for artificial insemination.

### Validation of the ss1815612719 polymorphism

PCR primers TTCAGTGCCAGGTTCATTGC and TTGGCTGGATGAGGTCAGTT were designed with Primer 3 [48] to scrutinize the ss1815612719 polymorphism by Sanger sequencing in two affected bulls and 97 unaffected artificial insemination bulls of the Swedish Red cattle breed. DNA was extracted from semen samples following standard protocols using proteinase K digestion and phenol-chloroform extraction. Genomic PCR products were sequenced using a 3730x1 DNA Analyzer (Applied Biosystems) and data were analyzed with the Variant Reporter v1.0 program (Applied Biosystems).

### Bioinformatic analysis of ARMC3

The ARMC3 protein sequence was obtained from ensembl (ENSBTAT00000061467) and the *ClustalW2* tool [49] was used for multiple species alignment. The annotation of ARMC3 protein domains was carried out using the *Simple Modular Architecture Research Tool* [50].

## Availability of supporting data

The data supporting the results of this article are included within the article and its additional files. Whole-genome sequencing data of a bull with the tail stump sperm defect were deposited in the European Nucleotide Archive (http://www.ebi.ac.uk/ena) under accession number PRJEB12739.

## Additional files

Additional file 1:
**Pedigree of three bulls with the tail stump sperm defect.** Red and blue color represents three affected bulls and their common ancestor. The drawn pedigree includes only obligate mutation carriers. (PNG 27 kb) Additional file 2:
**Sequence variants identified using the** ***SAMtools*** **software package that were compatible with recessive inheritance.** Grey background indicates 15 sequence variants that were not polymorphic among 1147 animals of the 1000 bull genomes project. Red color indicates a coding variant compatible with recessive inheritance. The functional consequence of the alternative allele was predicted using the *Variant Effect Predictor* from Ensembl (see Methods). (XLSX 61 kb) Additional file 3:
**Structural sequence variants that were compatible with recessive inheritance.** Grey background indicates an intergenic sequence variant that was not polymorphic among 1147 animals of the 1000 bull genomes project. (XLSX 36 kb) Additional file 4:
**Genotype distribution of 73 candidate causal mutations for the tail stump sperm defect in 1147 animals from the 1000 bull genomes project.** Alternate allele frequency and genotype distribution of 73 variants in 29 breeds (homozygous animals for the reference allele | heterozygous animals | homozygous animals for the alternate allele). Grey color indicates variants that were considered as candidate causal mutations. Red color indicates the deletion mutation in the coding sequence of *ARMC3*. (XLSX 49 kb)

## Abbreviations

AI: artificial insemination
ARM: armadillo
MMAF: multiple morphological abnormalities of the flagella
ROH: runs of homozygosity
SNP: single nucleotide polymorphism
