# Supplementary figures and images for: A frameshift mutation in ARMC3 is associated with a tail stump sperm defect in Swedish Red (Bos taurus) cattle
Source: BMC Genet. 2016 Feb 29;17:49. doi: 10.1186/s12863-016-0356-7 (PMC4770540; doi:10.1186/s12863-016-0356-7)

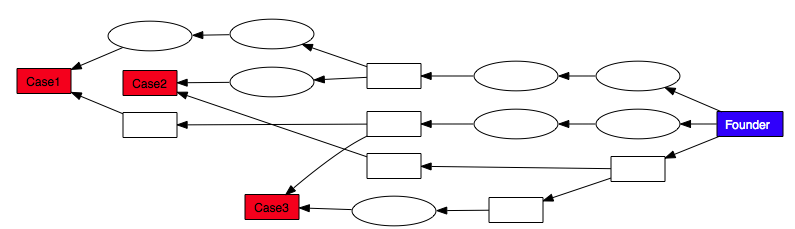

Supplement: Additional file 1: — Pedigree of three bulls with the tail stump sperm defect. Red and blue color represents three affected bulls and their common ancestor. The drawn pedigree includes only obligate mutation carriers. (PNG 27 kb) [file 12863_2016_356_MOESM1_ESM.png]
